# Detection of Long-Distance Transmission Events During the CSF Epidemic in Japan Using Whole-Genome-Sequence Data

**DOI:** 10.1155/tbed/5706784

**Published:** 2025-11-13

**Authors:** Takehisa Yamamoto, Tatsuya Nishi, Katsuhiko Fukai, Tomoko Kato, Yoko Hayama, Sonoko Kondo, Emi Yamaguchi, Ryota Matsuyama, Yuzu Kamata

**Affiliations:** ^1^Epidemiology and Arbovirus Group, Division of Transboundary Animal Disease Research, National Institute of Animal Health, National Agriculture and Food Research Organization, Tsukuba, Ibaraki, Japan; ^2^Exotic Disease Group, Division of Transboundary Animal Disease Research, National Institute of Animal Health, National Agriculture and Food Research Organization, Tsukuba, Ibaraki, Japan

## Abstract

Classical swine fever (CSF) is a highly contagious disease affecting domestic pigs and wild boars, posing a serious threat to the global swine industry. In Japan, CSF re-emerged on a pig farm in Gifu Prefecture in 2018, just 3 years after the country was declared CSF-free. The CSF virus (CSFV) was soon detected in neighboring wild boars and subsequently spread to adjacent areas, leading to further farm outbreaks. Given that long-distance transmission accelerates both spatial expansion and epidemic persistence, we aimed to identify such events during the current Japanese epidemic. Whole-genome sequences were generated for 100 farm isolates and 585 wild boar isolates collected through national surveillance. Putative ancestral strains were inferred for each isolate by comparing single-nucleotide variants (SNVs), and the great-circle distance to the nearest ancestral strain was considered the transmission distance. Six routes exceeding the 99th percentile of the distance distribution (182.2 km) were classified as long-distance transmission events: three involving farms and three involving wild boars. The sources of all these transmission events were identified as infected wild boars. The route to a farm in Okinawa Prefecture (January 2020) was linked to the illegal feeding of unheated food waste containing meat products. No specific sources were identified in the remaining two farm outbreaks. The three introductions into wild boar populations were most plausibly associated with anthropogenic activities, such as the movement of people or vehicles through infected habitats. To the best of our knowledge, this is the first study to comprehensively quantify long-distance CSFV spread across the entire course of the Japanese epidemic (2018–2024). Our findings will inform targeted control measures to prevent farm infections and the inadvertent spread of contaminated material to remote areas.

## 1. Introduction

Classical swine fever (CSF) is a highly contagious and often fatal disease affecting domestic pigs and wild boars caused by the CSF virus (CSFV), an enveloped, single-stranded, and positive-sense RNA virus of the genus *Pestivirus* (family Flaviviridae) [1]. Owing to its profound economic impact and capacity for rapid spread, CSF is a notifiable disease according to the World Organization for Animal Health (WOAH). National control and eradication programs for pig farms, therefore, depend on rapid diagnosis, movement restrictions, culling, and vaccination. Once CSF has been established in wild boars, eradication becomes challenging, as large-scale culling and movement control are impractical. In both pigs and wild boars, CSFV can be transmitted not only through direct contact but also via contaminated fomites, such as vehicles, clothing, tools, and even soil.

Japan attained disease-free status in 2015 after a decade-long eradication effort; however, the disease re-emerged on September 9, 2018, on a pig farm in Gifu Prefecture [2, 3]. Enhanced surveillance of wild boars soon revealed CSFV infections in local wild boars [2, 4]. As infections in wild boars increased, farm outbreaks also occurred in the same areas. By the end of May 2025, CSF had been confirmed in 41 of Japan's 47 prefectures: 99 farm outbreaks had been recorded, and approximately 420,000 animals from 178 farms and six related facilities had been culled.

**Figure 1:**
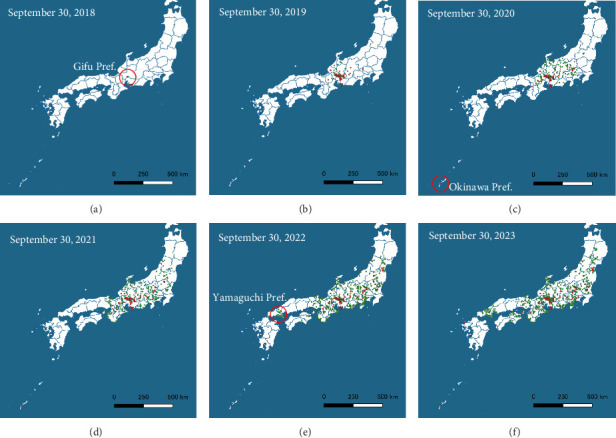
CSF epidemic since 2018 in Japan. Case farms (red) and infected wild boars (green) are shown on the annual map after the first case in September 2018. Red circles on map A denote the first case farm. Red circles on maps C and E indicate distant outbreaks referenced in the main text. Abbreviation: CSF, classical swine fever. Panels (A–F) show the distribution maps for September from 2018 to 2023, respectively.

In addition to contiguous spread, infections have periodically appeared in remote areas [5, 6]. For example, in January 2020, an outbreak occurred on a pig farm in Okinawa Prefecture, almost 1000 km from mainland Honshu (Figure 1C), and CSF-positive wild boars were detected in Yamaguchi Prefecture in March 2022 despite the apparent absence of infection in the intervening region (Figure 1E). Genomic analysis confirmed that the viruses isolated from both cases were descendants of an epidemic strain circulating in Japan, ruling out external introduction.

Long-distance spread into spatially isolated susceptible populations can greatly affect epidemic size and persistence [7, 8]. Consequently, many studies have elucidated the transmission routes of human pathogens—Ebola virus [9, 10], *Mycobacterium tuberculosis* [11], HIV [12], and SARS-CoV-2 [13–15], and animal pathogens, such as rabies [16, 17] and foot-and-mouth disease [18]. Identifying the timing and geography of CSFV transmission, particularly long-distance events, could support evidence-based interventions.

In this study, we aimed to analyze whole-genome sequences of farm and wild boar isolates to infer ancestral relationships, measure transmission distances, and identify long-distance transmission events during the Japanese CSF epidemic. Events exceeding the 99th percentile of transmission distances were examined in detail, and their implications for control strategies are discussed.

## 2. Methods

### 2.1. Sample Collection

Farm outbreaks: Blood and/or tissue samples (tonsils, spleens, and kidneys) were collected by prefectural Animal Health Service Centers (AHSCs) from clinically suspected farms and submitted to the Exotic Disease Branch, National Institute of Animal Health (NIAH). Complete genomes were obtained from 100 isolates representing outbreaks up to the 92nd farm case (May 27, 2024).

Wild-boar surveillance: Blood and/or tissue samples from wild boars that were captured or found dead were tested for CSFV via polymerase chain reaction (PCR) in AHSCs or in designated laboratories. PCR-positive samples were forwarded to the NIAH in consultation with the Ministry of Agriculture, Forestry, and Fisheries. Among > 4000 samples from 33 prefectures, 585 complete genomes were obtained on July 5, 2024.

### 2.2. Genome Sequencing

Viral isolation, reverse transcription PCR (RT-PCR), and next-generation sequencing were performed as described by Nishi et al. [19]. When direct PCR yielded insufficient products, viral isolation was performed using CPK cells. Viral RNA was amplified using four CSFV-specific primers. Libraries (~300 bp fragments) were prepared using the Ion Plus Fragment Library Kit and Ion Xpress Barcode Adapters (Life Technologies), followed by emulsion PCR on the Ion OneTouch 2 system (Life Technologies) and sequencing on an Ion Torrent PGM (Life Technologies). Reads were processed with Torrent Suite v5 (Life Technologies) (default settings; coverage threshold ≥ 15) to generate consensus sequences. Multiple sequence alignments were performed using MAFFT v7.311. An 11,826 nt region (5′ NTR to NS5B) devoid of indels was used for downstream analyses.

Among the GenBank entries, strain BJ2−2017 (MG387218.1) showed the highest similarity to the index-case isolate. To enhance single-nucleotide polymorphism (SNP) resolution, we created an outbreak-specific pseudo-reference genome (“artificial reference”) by reverting four private substitutions in the index isolate to ancestral nucleotides shared with BJ2-2017 [20–22]. Specifically, four positions were reverted relative to BJ2-2017: C7413T (NS4B), C7541T (NS4B), G9209A (NS5A), and A9400G (NS5A). This sequence approximates the most recent common ancestor (MRCA) and serves as a reference for all analyses.

### 2.3. Measurement of Transmission Distance

Selecting a trustworthy source for a newly detected strain requires more than choosing the nearest neighbor in space or genotype. Given that CSFV accumulates point mutations over time, any direct ancestor must (i) share all single-nucleotide variants (SNVs) observed in the descendant, and (ii) lack additional shared SNVs that are absent from the descendant. Shared SNVs refer to nucleotide substitutions present in two or more isolates within the dataset, in contrast to private SNVs, which are unique to a single isolate.

**Figure 2:**
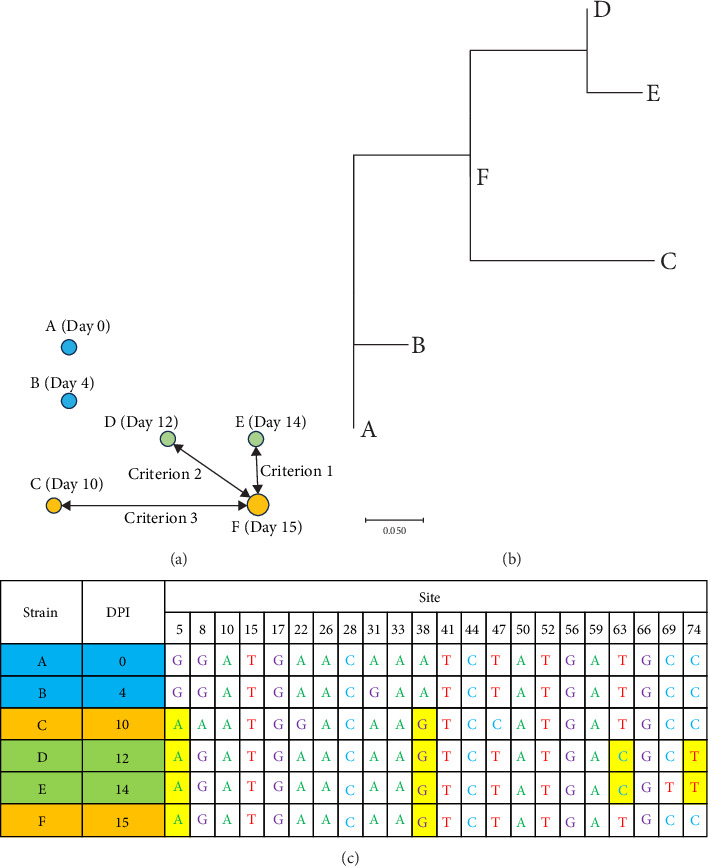
Key features of the criteria to identify a source strain of the questioned destination strain (F). (A) Geographical distribution of the strains (A–F). Days after the outbreak are shown in the parentheses. (B) Phylogenetic tree. (C) Single-nucleotide variants. Consensus sites were ignored. Key sites described in the main text are highlighted in yellow. DPI, days post infection of the first animal (A).

Illustrative criteria for identifying source strains are shown in Figure 2 for destination strain F (detected on day 15) and three earlier isolates, C, D, and E:

Criterion 1: “Geographic proximity” selects E, the spatially nearest isolate, regardless of its mutational profile.

Criterion 2: “Genetic proximity” selects D, the isolate with the fewest SNV differences from F.

Criterion 3: “Ancestral compatibility” selects C, the isolate that carries the complete shared SNV set of F but no additional shared SNVs. Only C satisfies a parsimonious ancestor–descendant relationship; D and E would require back-mutation or unsampled intermediates.

We therefore adopted criterion 3.

**Figure 3:**
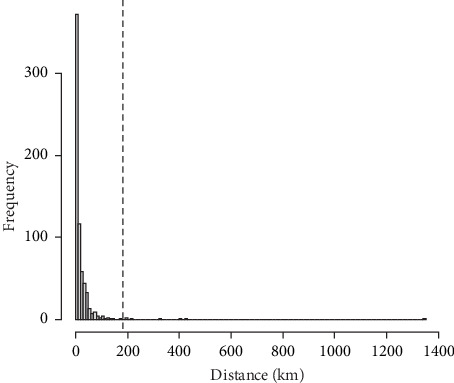
Distribution of transmission distance in the CSF epidemic in Japan since 2018. The 99th percentile is shown in a dashed line. Abbreviation: CSF, classical swine fever.

Consequently, the algorithmic implementation for identifying long-distance transmission events is as follows:

1. SNV catalog: All 685 genomes were aligned to an artificial reference, and a binary SNV presence/absence matrix was generated.
2. Sequential grouping [5]: Starting from a single group, isolates were recursively partitioned using discriminatory SNVs until each group contained genomes with identical SNV profiles.
3. Source-candidate set: For a destination isolate *x* sampled at time *t*_*x*_, candidate sources were those isolates that (a) belonged to the deepest shared SNV group with *x* and (b) were sampled at *t* < *t*_*x*_.
4. Distance computation: The geodesic distances between the coordinates of *x* and each candidate were calculated using the distGeo function (Geosphere package), which computes the shortest path on an ellipsoid, and the minimum value was recorded as the transmission distance for *x*.
5. Long-distance definition: The empirical distribution of the transmission distances (Figure 3) was strongly right-skewed. Distances exceeding the 99th percentile were classified as long-distance events and examined individually.

This procedure combines phylogenetic parsimony with spatial realism, thereby reducing the risk of incorrectly assigning sources with private mutations or those detected after their putative descendants.

## 3. Results

**Figure 4:**
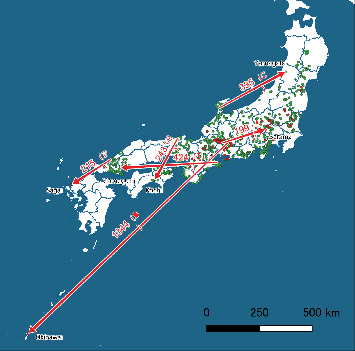
Long-distance CSF transmission events in Japan since 2018. Numbers on the arrows denote distances in kilometers, with transmission IDs in parentheses (Table 1). Circles indicate farm cases (red) and infected wild boars (green) for which whole genome sequence data were available. The names of the destination prefectures are shown near the arrowheads. Abbreviation: CSF, classical swine fever.

The distribution of the transmission distances (Figure 3) was strongly right-skewed, with a mean of 20.9 km and quartiles at 2.3, 8.7, and 21.9 km; the 95% range spanned 0–99.7 km. Seven candidate routes were within the top 1% of the distances, exceeding the 99th percentile threshold of 182.2 km. Two of these originated from Ishikawa-derived wild-boar strains and led to Yamagata—one to a pig farm (case 60), and the other to a wild boar. Further inspection revealed that, although the pig farm isolate was detected earlier, it was genetically downstream of the wild boar isolate, suggesting delayed detection of the latter. We therefore adopted the route to the wild boar as the more plausible transmission event, and excluded the newer, genetically downstream route to the pig farm, leaving six high-confidence long-distance transmission events (Table 1; Figure 4). The distances ranged from 199 to 1344 km. Three routes (A, B, and F) ended in farms, and three (C, D, and E) ended in wild boar populations. All sources were inferred to be wild-boar isolates.

## 4. Discussions

When infectious disease transmission occurs discontinuously across distant locations, predicting the spread and protection of disease-free areas becomes challenging. Therefore, understanding long-distance transmission is essential. Using whole-genome sequence data from the Japanese CSF epidemic (2018–2024), we inferred source–destination relationships and measured transmission distances, identifying six events above the 99th percentile.

### 4.1. Transmission to Farms

Three long-distance transmission events (A, B, and F) ended on the pig farms. Transmission B to Okinawa Prefecture, 1344 km from its estimated source in Gifu Prefecture, occurred without any detected infection in Okinawan wild boars, suggesting that CSFV was introduced directly into the farm. An epidemiological investigation revealed that food waste containing meat products is illegally fed to pigs, which is the most likely route [6]. The carcass of the wild boar that yielded the source strain was disposed of after testing and could not have caused the outbreak; rather, its detection indicated prior circulation of the strain in the area where the infected wild boar was found in Gifu, highlighting the possibility that they may have entered Okinawa via contaminated meat products. Given that farms in the source region had already been vaccinated, any CSFV incursion could have remained transient and clinically inconspicuous. Pork from such a farm might have entered the food-waste stream and become a contaminated meat product fed to pigs in Okinawa.

For the other two transmission events (A and F), neither swill feeding nor other specific risk factors were identified during the on-site investigations, and the precise introduction routes remain unknown. Nevertheless, evidence of CSFV spread over 1300 km emphasizes the need for stringent control of unheated swill feeding on pig farms.

### 4.2. Transmissions to Wild Boars

The remaining three transmission events (C, D, and E) ended within wild-boar populations. Transmission C to Yamagata Prefecture was first identified with an almost identical route leading to a nearby pig farm (case 60). However, the route was removed by diagnosis as misclassified owing to the delay in detecting ancestral wild boar infection in the same area. Therefore, we inferred that CSFV first entered the local wild boars and subsequently spilled over to the farm. For transmissions D and E, no farm infection has been identified near the destination wild boars, suggesting a direct introduction into wildlife. Although adult male boars can roam 50–250 km during the rutting season [23], the observed distances (243–424 km) and the absence of intermediate detections are difficult to reconcile with natural movements alone. Therefore, anthropogenic dissemination through contaminated vehicles, equipment, or soil is plausible [6, 24]. To reduce this risk, the Ministry of Agriculture, Forestry, and Fisheries and the Ministry of Environment have issued guidelines on washing and disinfecting clothing, footwear, and vehicle tires after visiting forests or parks where infected wild boars may be present [4].

### 4.3. Methodological Considerations

Long-distance transmission of human diseases is often detected through contact tracing [8, 25] or the phylogenomic reconstruction of pathogen movement [11, 13, 14, 26]. Given that formal contact tracing is infeasible in wildlife, genome sequencing provides an indispensable surrogate for mapping transmission pathways in animal populations [27–29]. Therefore, the comprehensive whole-genome dataset assembled from wild boar and pig farms enabled a robust reconstruction of CSFV movements both within and between host species.

### 4.4. Sampling Bias

All farm outbreaks confirmed to date have been subjected to whole-genome sequencing. Nevertheless, small-scale infections may have been missed after routine vaccination commenced in October 2019. Such undetected incidents would probably have involved limited within-farm spread, and consequently, a low probability of onward transmission. In wild boars, the sampling intensity varies markedly among prefectures, being the weakest in regions that have not yet reported infections [4]. Of the > 4000 PCR-positive samples, 585 genomes were analyzed in this study, leaving the possibility that unsampled viral lineages exist. The fact that the first isolate detected in each prefecture was always prioritized for sequencing increased the likelihood that long-distance introductions to new areas would be promptly detected and included in the analysis. Moreover, no additional isolates that were more closely related to the putative sources emerged in the intermediate areas during the 12 months following the last long-distance event (F), lending further credence to our inferences. Nevertheless, because the strain selection for sequencing was partly subjective, the absolute frequency of long-distance transmission may be inflated. Accordingly, the route list presented here should not be used to estimate the full-transmission-distance distribution [30, 31].

### 4.5. Implications for Control Policy

Long-distance jumps amplify the epidemic size and prolong persistence, thereby undermining conventional containment strategies [32–34]. Our finding that at least one such jump (transmission B) was precipitated by the misuse of unheated swill and that several others were probably driven by unchecked human or vehicular movements underscores the need for stringent enforcement of existing biosecurity regulations. Notably, the prohibition on feeding unprocessed swill to pigs must be rigorously policed, and travelers entering or traversing infected habitats should be reminded through signage, media campaigns, and targeted outreach to clean and disinfect clothing, footwear, and vehicle tires. Although preliminary summaries of these findings were provided to the Japanese authorities during official briefings on epidemic control, the present article constitutes the first systematic, peer-reviewed study to rigorously quantify long-distance CSFV transmission events across the Japanese epidemic and interpret their epidemiological implications.

## 5. Conclusions

Six long-distance CSFV transmission events were identified during the 2018–2024 Japanese epidemic: three on farms and three in wild boars, all traceable to wildlife sources. Evidence highlights two principal risk pathways: the illegal use of unheated swill, which has been implicated in farm outbreaks, and the inadvertent transport of contamination by people or vehicles moving through forested habitats. Therefore, effective prevention hinges on two strategies: (i) strict enforcement of swill-feeding regulations at farms and (ii) rigorous cleaning and disinfection of clothing, equipment, and tires when entering or leaving mountainous areas. Continued whole-genome surveillance is essential for detecting future long-range introductions and for guiding rapid control measures.

## Figures and Tables

**Table 1:** List of long-distance transmission events identified during the CSF epidemic in Japan.

Transmission ID	Destination strain				Source strain				Distance (km)
	Sampling date	Case/WB	Accession #	Location	Sampling date	Case/WB	Accession #	Location	
A	12-09-2019	Case (#41)	LC583657	Chichibu, Saitama	17-01-2019	WB	LC583616	Inuyama, Aichi	199
B	06-01-2020	Case (#52)	LC583679	Uruma, Okinawa	13-02-2019	WB	LC583663	Mizunami, Gifu	1344
C	04-05-2021	WB	LC881300	Tsuruoka, Yamagata	03-03-2021	WB	LC881252	Shika, Ishikawa	335
D	13-03-2022	WB	LC881394	Iwakuni, Yamaguchi	29-07-2021	WB	LC881625	Ise, Mie	424
E	07-09-2022	WB	LC881422	Kami, Kochi	07-06-2022	WB	LC881596	Tango, Kyoto	243
F	30-08-2023	Case (#88)	LC881155	Karatsu, Saga	07-11-2022	WB	LC881600	Masuda, Shimane	215

## Data Availability

The datasets analyzed in this study can be found in the GenBank database (accession numbers: LC583562–LC583718 and LC881125–LC881651). A complete list of these strains is provided in Supporting Table 1.

## Supporting Information

Supporting Information Table S1: CSFV isolates obtained from 2018−2024 epidemic in Japan. The table contains a brief description of each sample, sample type (case/wild boar), date of sampling, location, strain name, tip name, and GenBank accession number.
